# Living through a Global Pandemic: A Cross-Sectional Study on the Psychological Resilience of the University Population in Iran

**DOI:** 10.3390/ijerph20064844

**Published:** 2023-03-09

**Authors:** Fereshteh Ahmadi, Önver Andreas Cetrez, Saeid Zandi

**Affiliations:** 1 Department of Social Work and Criminology, Faculty of Health and Occupational Studies, University of Gävle, 80176 Gävle, Sweden; saeid.zandi@hig.se; 2 Department of Psychology of Religion, Faculty of Theology, Uppsala University, 75120 Uppsala, Sweden; onver.cetrez@teol.uu.se

**Keywords:** academia, academicians, academics, collective trauma, coping, COVID-19, crisis, health, higher education, resiliency

## Abstract

Aims: This study aimed to describe and understand the individual and social dimensions of resiliency among Iranian academics as professionals during the early wave of the ongoing pandemic. Furthermore, we aimed to emphasize the cultural context in our analysis. Method: A cross-sectional survey design was adopted. We used convenient sampling, administered through an online survey, among academics at Iranian universities (*n* = 196, 75% women). We employed the CD-RISC 2 instrument, items on life meaning, and a modified version of Pargament’s RCOPE instrument (Meaning, Control, Comfort/Spirituality, Intimacy/Spirituality, and Life Transformation). Results: The results revealed a strong level of resilience among men (*M* = 5.78) and women (*M* = 5.52). Self-rated health was rated as excellent, very good, or good among a majority (92%) of the participants, more so among men. Family was one of the factors that most strongly gave life meaning, followed by friends, work/school, and religion/spirituality. There was a strong correlation between self-rated health and life as part of a greater whole, being alone, and listening to the sounds of the surrounding nature. Conclusions: Both personal and social levels of resilience and meaning-making are seen in the results, with an ability to balance between obstacles and resources. Cultural practices are interdependent, which also include the individual and social dimensions of resiliency and meaning-making.

## 1. Introduction

The world has been experiencing mass trauma from COVID-19 since the beginning of the pandemic. The worldwide public health crisis caused by severe acute respiratory syndrome coronavirus-2 (SARS-CoV-2) affected countless individuals in all countries [1,2]. On 19 February 2020, it was announced that COVID-19 reached Iran in terms of general community transmission. At the beginning of the pandemic, Iran ranked third in the number of people suffering from the disease, after China and South Korea, and second in relation to the mortality and recovery rate [3]. For Iranians, as for people in many other nations, this was their first experience with a health crisis of an indiscernible agent, resulting in high levels of uncertainty and detrimental aftereffects on psychological health. Iran has more than 2600 academic institutions, universities and colleges, which means that during the pandemic, more than four million people were studying/working from home.

Negative psychological consequences caused by the pandemic and lockdown were found among university staff and students [4,5]. A study found that stress, anxiety, and depression were significantly associated with fear of infection, financial uncertainty, inadequate food supply, absence of physical exercise, and limited recreational activity among students in Bangladesh [6]. Moderate to extremely severe levels of anxiety, depression, and stress were also reported among staff and students in Spain [7]. However, as Counted et al. rightly point out, the specific pathways by which a public health crisis impacts psychological well-being might be more devastating for individuals with pre-existing mental health problems [8].

A survey during the coronavirus pandemic in Iran revealed that half of the participants reported serious anxiety, a fifth reported suffering from moderate anxiety, and a third suffered from low anxiety [9]. The same survey also revealed that the families’ economic resilience was low and that a sixth of the families reported an increase in tensions due to staying at home. Furthermore, more than half reported an increase in tensions between couples and almost half an increase in tensions between parents and children.

Counted et al. examined hope and religious coping as protective resources for well-being among Colombian students and South African participants living under lockdown conditions [8]. The findings revealed higher levels of positive religious coping and lower levels of negative religious coping associated with higher levels of well-being. Furthermore, hope yielded a positive association with well-being. A multiple regression analysis revealed that the relation between hope and well-being was partially moderated by religious coping. Even so, when hope was low, well-being was higher when positive religious coping was higher, and negative religious coping was lower. The authors refer to previous research, stating that people engaging in positive religious coping strategies in times of crises are able to reinterpret the circumstances more positively, believing that a divine purpose is at work, and thus keep a sense of control [8]. The authors also conclude that in contexts where resources are limited in times of crises and lockdowns, people may look internally for hope and a higher power to adapt to stressors; thus, religion comes into the foreground.

A study in Italy showed that people who reported a contagion in their family showed higher frequencies of attending religious services and prayer, especially for those who were previously religiously socialized [10]. In other words, and referring to earlier research, Molteni et al. state that religion as a coping mechanism is efficient only if it is already part of an existing orienting system or cultural toolkit [10].

An Iranian study conducted by the Research Center for Culture, Art and Communications revealed that a fifth of respondents listened to music, read books, or talked to family members during quarantine to relax and decrease tension [11].

Some studies conducted research on psychological resilience during the COVID-19 pandemic [12,13]. During the first wave of COVID-19, a U.S. study showed that psychological resilience was significantly lower when compared to earlier normative data, as measured by the CD-RISC scale [14]. Further, lower scores on the CD-RISC were associated with worse mental health outcomes (depression, suicidal ideation, and severe anxiety) [15]. The same study revealed an association between lower resilience and greater worry about the effects of COVID-19. Interestingly, the study by Killgore et al., using multiple linear regression, predicted greater resilience in relation to social factors, such as daily outdoor activities, social support from family, friends, and close significant others, and prayer. Other studies showed an increased prevalence of anxiety and depression levels among Chinese and Swiss adolescents associated with the COVID-19 pandemic [16].

Age differences in psychological resilience were found in a Chinese study, also during the first wave of COVID-19, where older participants (>55 years) showed higher resilience than the younger group (<18 years), possibly due to life experience [17]. The same study also revealed lower CD-RISC scores for the depressed group, when compared to those who were not depressed.

Among Swedish academics, several secular existential coping methods appeared as the most common: among these, nature, followed by listening to the sounds of surrounding nature, thinking of life as part of a greater whole, walking/being active outdoors, being alone, and thinking of an internal spiritual force exist [18]. Cetrez et al. investigated the individual and collective dimensions of resilience among academic community members in Sweden [19]. The findings revealed a strong level of personal/individual resilience among men and a level just below strong among women. By age group, those 35-49 years old showed strong resilience. Family was the dominant social/collective resilience factor, followed by friends, nature, work/school, and, lastly, religion/spirituality. There was a positive and significant correlation between self-rated health and personal/individual resilience and positive but weak correlations and negative significant correlations between personal/individual resilience and religious coping methods. A non-COVID-19 study among university students in Ethiopia showed that a higher level of resilience was related to higher coping strategy, with a strong correlation between psychological resilience and task-oriented, emotion-oriented, and avoidance-oriented coping [20]. Research also shows a positive association between resilience and better psychological and physical outcomes [21].

## 2. Theoretical Framework

In the 1980s, *resilience*, a psychological term, was synonymously used for “the ability of individuals to recover from exposure to chronic and acute stress” [22] (p. 13). After further development, resilience was seen as the coping behavior of individuals in the face of great adversity, thus “[r]esilience refers to a class of phenomena characterized by *good outcomes in spite of serious threats to adaptation or development*” [23] (p. 228, emphasis in original). Hence, the individual is expected to exhibit great hardiness, durability, and adaptation in order to master episodes of difficulties and to avoid surrendering to despair. In this sense, “resilience is part of what has helped humans survive” [24] (p. 18) and “what helps them to move on and regain stability and productivity” [25] (p. 3). Despite this risk context, adolescents show the ability to remain healthy, thus indicating that resilience is a dynamic process with protective factors to buffer risks [16].

Researchers wanted to investigate what allows individuals to cope with situations of great risk. Early studies on human development focused predominantly on personal characteristics, such as the individual’s abilities, strengths, motivation, traits, and talents, as well as genetic predispositions, as factors influencing an individual’s personal adaptation skills. Already in 1979, psychologist Urie Bronfenbrenner criticized this one-dimensional approach and highlighted the shortcomings in not acknowledging the profound importance of the environment’s influence on the individual. Although extra-individual factors were accounted for, they were not centered as the focus of the research because “personal qualities” were regarded “as the *sine quo non* of developmental outcomes” [22] (p. 15, emphasis in original) within this individualistic approach. Opposed to this, further studies of resilience established conceptualizations of this phenomenon by looking more in-depth at structural factors and by focusing on “how the fabric of a society impacts individual mental health trajectories.” [26] (p. 369).

Similarly, criticism of earlier resilience research points out the overemphasis on the individualized nature of adaptation, typical of western or mainstream populations, and the lack of sensitivity to community and cultural factors in contextualizing resilience practices [27,28]. Thus, Summerfield highlights the cultural differences in resilience concepts [22] (p. 341):

*The cultural emphasis [among non-Western people] is on dependency and interdependency rather than the autonomy and individualisation on which many western ideas about mental injury are predicated.*

Our knowledge of processes of resilience in contexts other than western cultures, not least in crises such as COVID-19, is limited. A culturally and contextually sensitive definition of resilience presented by Ungar indicates both the process of navigation and negotiation, and is useful for this study:

*In the context of exposure to significant adversity, whether psychological, environmental, or both, resilience is both the capacity of individuals to navigate their way to health-sustaining resources, including opportunities to experience feelings of well-being, and a condition of the individual’s family, community and culture to provide these health resources and experiences in culturally meaningful ways.*

[28] (p. 225)

Ungar’s constructivist approach emphasizes the significance of social relations, thus challenging the dominant discourse of pathology and health, arguing that judgments about normalcy, deviance, and health held by researchers may be opposite to those held by participants [27]. Furthermore, resilience should be regarded as an interactive two-way process, which is nurtured by external stimuli and at the same time depends on the individual’s internal perceptions [22]. Thus, this constant negotiation between the individual and their environment(s) is driven by opportunities and obstacles, which the individual is presented with and to which they respond. The opportunities encompass resources (social, cultural, psychological, physical), which need to be available and accessible for the individual [22].

Additionally, the resources must be meaningful to the individual, in order to help enhance resilience. “‘Meaning making’ designates the process by which people interpret situations, events, objects, or discourses, in the light of their previous knowledge and experience” [29] (p. 1809). This meaning is culturally specific and refers to the fact that the meaningfulness needs to be evident to the individual and needs to match their needs which, e.g., depends on their socialization, respectively “their previous knowledge and experience” [29] (p. 1809). Thus, meaning links to the system, which signals to individuals and communities the importance of certain factors within their life, or at least for certain areas in their lives such as, e.g., well-being. As a consequence, this meaning-making determines the decision for and against specific resources, as well as the ability to determine what is meaningful, i.e., what is needed for positive development. Ungar goes on by pointing out that meaning-making guides people towards what they perceive as purposeful actions and “to which resources (opportunities) they value and access” [22] (p. 22). Furthermore, his argument is that resources provided depend on the meaning that is attributed to them, usually indicated by the dominant culture within a specific socio-cultural, socio-historical, and time-specific framework. Thus, “the opportunities that we create” are always bound by context [22] (p. 22). Ungar continues by stating that ecology and individuals find themselves in an interactive and reciprocal relation, whereas externally available and accessible resources mobilize personal strengths internally. Once opportunities (such as, e.g., support systems) are offered, the individual can make use of them and draw from them by building up their own ability to cope. This also entails the capacity to negotiate for resources, implying the active nature of individuals to stand up for their needs and for the resources they feel should be provided.

Some central support systems for resilience are family and community. The family context offers economic resources, shared beliefs and values, affectionate rituals, traditions, support systems, and positive self-esteem, among others [22]. Community, in turn, contributes to social hope, attachment and belonging, social support and connectedness, collective goals, and rituals, among others [22,25]. Both family and community are linked to belief systems, organizational patterns, and communication processes [22]. The role of family is also highlighted by Ahmadi et al. in Iranian culture [30] (p. 13):

*There are some mainstays of Iranian society, such as the importance of family, the proud adherence to local culture and traditions, and the tendencies toward post-modern ways of life and ways of thinking that influence the choice of coping strategies.*

Our approach to resilience and meaning-making necessitates a working definition of culture. The definition provided by cultural psychologist Marsella refers to internal and external behaviors, as well as meanings, encompassing all human processes [31] (p. 657):

*Culture is shared learned behavior and meanings that are socially transferred in various life-activity settings for purposes of individual and collective adjustment and adaptation. Cultures can be (1) transitory (i.e., situational even for a few minutes), (2) enduring (e.g., ethnocultural life styles), and in all instances are (3) dynamic (i.e., constantly subject to change and modification. Cultures are represented (4) internally (i.e., values, beliefs, attitudes, axioms, orientations, epistemologies, consciousness levels, perceptions, expectations, personhood) and (5) externally (i.e., artifacts, roles, institutions, social structures). Cultures (6) shape and construct our realities (i.e., they contribute to our world views, perceptions, orientations) and with this, our concepts of normality/abnormality, morality, aesthetics, and a number of arbiters of life.*

Thus, when using resilience in this article, we refer to intra- and interpersonal practices, as well as the environment’s influence on individual and collective behaviors and meanings.

## 3. Aims

As little research attention has been paid to the dimensions of resilience among the Iranian university community during the current global epidemic, the aim of this study is to relate gender and age to the resiliency, health, and life meaning, of individuals working or studying in academic settings in Iran, who are in a context of social isolation and challenged by COVID-19. The independent variables are gender (men/women) and age (<25/25–35/>35 years). The dependent variables are defined as resiliency (adapt/bouncing back), self-assessed health status, importance of family and friends, religion/spirituality, and life meaning. We also aim to test the relationship between self-assessed health status and coping methods.

The research questions guiding this study are:

- Q1. How strongly do academics in Iran rate their level of resiliency (measured using the CD-RISC 2, and disaggregated by gender and age group)?
- Q2. How strongly do academics in Iran rate their health (disaggregated by gender and age group)?
- Q3. How strong are family, religion/spirituality, work/school, and friends as life meaning factors among academics in Iran?
- Q4. What is the correlation between self-rated health status and CD-RISC and coping methods, respectively, among academics in Iran?

## 4. Materials and Methods

A quantitative research design was employed to conduct this cross-sectional study.

### 4.1. Sampling

The target group consisted of academics active in Iranian universities, including both staff/faculty members and students. For this study, we found a list-based sampling frame, with a convenient sampling method most useful, as the academic groups were homogeneous and e-mail addresses were available [32]. The inclusion criteria were university staff, students, full or part-time, at any Iranian university or college.

### 4.2. Procedure

For data collection, we used the Iranian online survey maker (www.cafepardazesh.com, accessed on 20 December 2022). The link was e-mailed to faculty members, students, and other university staff on 30 May 2020. The e-mail first presented an invitation letter before participants were asked to give their consent and answer the questions. The online survey closed on 9 June 2020. At that time, 210 women and men working or studying at different universities had completed the questionnaire. Some questionnaires were excluded due to missing data, leaving 196 questionnaires for analysis. Table 1 presents the demographics of the participants.

As seen in Table 1, sample characteristics, the majority of participants were women. For educational level, the vast majority were at university level or equivalent, and most were born and resided in Iran. Among the respondents, 39 percent were distance-learning students, and 22 percent campus students. Furthermore, 25 percent were employed full-time and 14 percent part-time. The majority were single, 56 percent, followed by married, 38 percent, and very few were divorced or engaged. More than 3 in 4 did not have children. Almost 58 percent lived in the capital, 36 percent in a medium–large city or small town, which was close to a large city, and very few lived in a small town, far from a large city.

### 4.3. Measures

The survey comprised items linked to the theoretical framework of resilience. Resilience was gauged using two items from The Connor–Davidson Resilience Scale (CD-RISC 2), the ability to adapt to changes and bouncing back after illness [14,33]. Respondents who score 6+ indicate a strong level of resilience. Items linked to meaning of life, in order to document the individual and cultural dimensions of resilience, were also included. To evaluate health, we included items on self-perceived health. Furthermore, we included demographic items on gender, age, educational level, employment, and place of residence. A modified version of Pargament’s RCOPE instrument was used (Meaning, Control, Comfort/Spirituality, Intimacy/Spirituality, and Life Transformation). The RCOPE has a Cronbach’s Alpha value of 0.794 (high level), and includes 15 items, rated on a 4-point Likert scale ranging from 0 (“Never”) to 3 (“Always”), plus 9 background items. The instrument was validated for language and content in earlier studies [18,19]. Content and concepts were also adjusted to fit the Iranian cultural context, where mosque, religious leader, and Allah replaced terms such as church, priest, and God.

### 4.4. Data Analysis Methods

Calculations such as cross tabulations (by gender and age group) and Pearson’s correlation were performed using SPSS^®^ Statistics Version 27 (SPSS Inc., Chicago, IL, USA). The sample has not been weighted to reflect the actual academic populations it represents.

### 4.5. Ethics

Following the World Medical Association Declaration of Helsinki [34], an application for ethical approval was handed in to the Swedish Ethical Review Authority, as the data are being analyzed and preserved in Sweden (Reg. No. 2020/02368 9). For the data gathering in Iran, an internal academic group in Iran studied the research project and questions and approved them. The ethical guidelines for the study were based on the Economic and Social Research Council’s ethical principles for humanities and social science research (ESRC Framework for Research Ethics. Updated January 2015. Available online: http://www.esrc.ac.uk/files/funding/guidance-for-applicants/esrc-framework-for-research-ethics-2015/, accessed on 20 December 2022). We clarified with potential respondents, together with an initial letter of information and asking for consent, that participation was voluntary, data would be treated with care, and results of the study would be published on a group level only.

## 5. Results

### 5.1. Resilience

**Figure 1:**
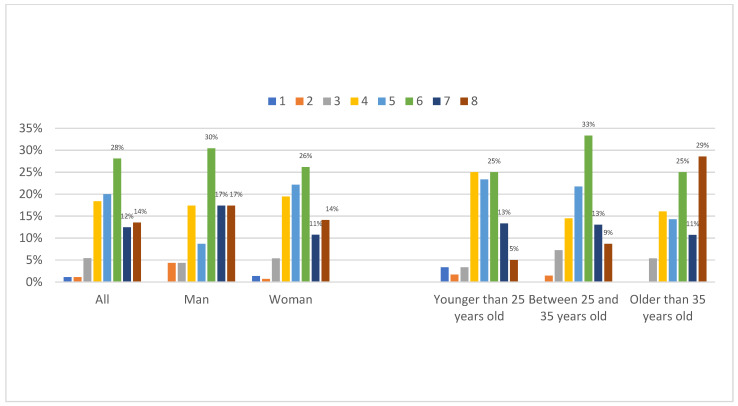
Resilience (CD-RISC 2), by gender and age group, by percentage. (Note. Scaling from 0 ‘Not true at all’ to 8 ‘True nearly all the time’).

Figure 1, CD-RISC 2, displays the capacity to recover from a disease such as COVID-19, in which the academics in Iran were asked two questions—if they can adapt when changes occur and if they tend to bounce back after illness, injury, or other hardships—on a scale from 0 (not true at all) to 4 (true nearly all the time). This figure reflects the merging of responses from both questions, creating an index (0–8). Men (*M* = 5.78) showed slightly higher resilience than women (*M* = 5.52), but the difference was not significant. Fifty-four percent (those ranging from 6 to 8 on the index) had high resilience. It was higher for men, but also higher for those older than 35 years. As many as 29 percent of the older group had an index value of 8.

### 5.2. Self-Rated Health

**Figure 2:**
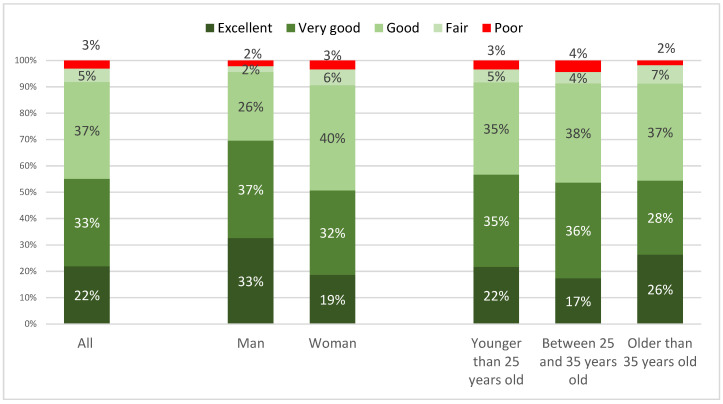
Self-rated health during COVID-19, by gender and age group, by percentage.

As seen in Figure 2, self-rated health, 55 percent of the academics in Iran say their health is excellent or very good. Another large share, 37 percent, say their health is good. That accounts for 92 percent who say their health is at least good. Only three percent say it is poor. Men more often claim that their health is excellent or very good, 70 percent versus 51 percent for women. Only very small differences occur across ages, as 57 percent of the young claim their health is excellent or very good, which, respectively, is 53 percent for 25–35 year olds, and 54 percent for those older than 35 years. Notably, the older group says that their health is excellent more often.

### 5.3. Life Meaning

**Figure 3:**
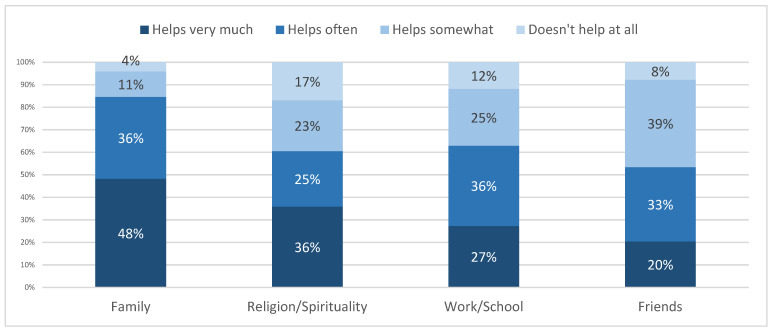
Giving meaning to life during COVID-19, by percentage.

As seen in Figure 3, items that give life meaning, about 9 out of 10 academics in Iran claim that their family, religion/spirituality, work/school, and friends give them meaning in life. In ranking order, as many as 96 percent (the percentages relate to those that ranked items as ‘helps very much,’ ‘helps often’, and ‘helps somewhat’) say their family has helped them, the highest proportion responding ‘very much’; 92 percent are helped by friends, the highest proportion responding ‘somewhat’; 88 percent are helped by work/school, the highest proportion responding ‘often’; and 83 percent that they have been helped by their religion or spirituality, the highest proportion responding ‘very much’.

### 5.4. Self-Rated Health, Resilience and Coping Methods

**Figure 4:**
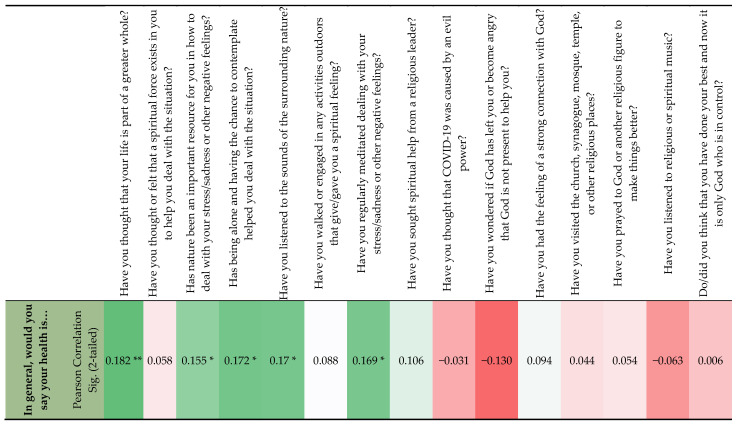
Correlation between self-rated health and frequency of different coping methods. Note. ** Correlation is significant at the 0.01 level (2-tailed); * Correlation is significant at the 0.05 level (2-tailed); Dark green shows the strongest correlation, and dark red the weakest correlation.

Figure 4 displays the correlation between self-rated health and the frequency of different coping methods, the higher the usage of different coping methods, the higher the self-rated health, or vice versa. The correlation is strongest between self-rated health and life as part of a greater whole, followed by being alone, listening to the sounds of the surrounding nature, regularly meditating, and nature as an important resource.

The CD-RISC correlated significantly with the coping methods “life is part of a greater whole” (r = 0.196, *p* = 0.01), “nature as an important resource” (r = 0.224, *p* = 0.01), “being alone” (r = 0.252, *p* = 0.001), “listening to the sounds of nature” (r = 0.159, *p* = 0.05), “engaging in outdoor activities” (r = 0.172, *p* = 0.05), “meditating” (r = 0.155, *p* = 0.05), “wondering if God has left me” (r = −0.149, *p* = 0.05), and “feeling a strong connection with God” (r = 0.149, *p*= 0.05).

There was also a positive and significant (r = 0.210, *p* = 0.01) correlation between self-rated health and CD-RISC, the higher the CD-RISC score, the more positively health was rated, or vice versa.

## 6. Discussion

Though we did not specifically measure the correlation between resilience and mental health, as done in other studies [15,16,21], our study did reveal a positive and significant correlation between resilience and general self-rated health among the Iranian university community.

Similarly to Song et al. [17], though using different age group categories, our results revealed a stronger resilience level among older age groups.

Similar to the study by Counted et al. [8], we also found that positive religious coping methods were correlated with positive health and resilience level.

Listening to music was not a strong coping method in our study, as seen in the results from the Research Center for Culture, Art and Communications [11]. However, this may be due to the design of our question, as music pertained to only religious or spiritual music. Listening to the sounds of the surrounding nature, which in its right is comparable to music, was on the other hand found to be a strong coping method among the Iranian academic community.

Reflecting back on the research questions of this study, we see that CD-RISC mirrors the individual dimension of resilience (ability to bounce back and adapt). Here, Iranian academics showed a close to strong resiliency level, with no significant differences found between gender and age (Q1). A clear majority rated their health as good (or higher); this was stronger among men, but no difference was found between ages (Q2). Thus, as the results for Q1 and Q2 show, the participants demonstrate good resiliency outcomes, in spite of the serious COVID-19 threat. For the community and dependency dimensions of resilience, which act as sources of support, our results revealed that family is the strongest for life meaning, followed by friends, work/school, and religion/spirituality (Q3). Having family as the main resource links well with the importance of family in Iran [30], the importance of the environment’s influence, and the importance of structural factors for the outcome of individual health. These findings also reiterate the importance of paying attention to community and cultural factors in resilience practices, rather than overemphasizing the individual factors [27,28]. Dependency rather than autonomy becomes a more important resilience indicator, which is not well reflected in the instruments of resilience.

Here, it is worth noting how teleworking mandated by the government during the coronavirus epidemic allowed work to be combined with household chores and childcare, which ultimately improved the balance between work and family. Academics then had more time to spend with family members, and therefore, family and family care became the most accessible source for meaning-making and resilience. This is more understandable in the context of the COVID-19 situation where almost no other form of face-to-face community (e.g., sports clubs, friends/colleagues network, etc.) was available; in other words, the individual found no better or more reliable thing to resort to than family. Family provides help and a sense of peace for its members. This may be fortified by the Iranian-specific devotion and dependence on family. As Ahmadi and Ahmadi mention [35] (p. 222):

*In the configuration of the identity of Iranians, characterized by the ideas of other-identification and negation of individuality, family relationships and ties of friendship play important roles. Actually, the alter-ego type of definition of family members and of friends is essential in Iranian culture, as it is in other Islamic cultures.*

In this context, one can witness a pattern of traditional family relationships with collectivist orientation that stresses security under family protection. This is unlike the western countries, where the dominance of the modern individualistic patterns of behavior has given rise to an individualistic interpretation of the roles of the individual as a member of the family. In Iranian culture, individuals learn from their parents the importance of putting the needs of the family before their own [36].

Our results also reflect the two dimensions of meaning-making, the individual and social. The social, community, or structural dimension can be found in the strong correlation between self-rated health and seeing life as part of a greater whole, nature, and outdoor activities. The individual dimension is reflected in the strong correlation between self-rated health and being alone, listening to nature’s sounds, meditation, and the connection with God (Q4). In sum, Ungar’s definition of resilience [28], being a capacity of both individual and collective factors, both internal and external processes, expressed and interpreted in a meaningful way, is useful in understanding the context for this study. However, for future analysis of resilience and COVID-19 among academics in Iran, we need to more closely analyze how structural resources are interpreted in context and what meaning is attributed to these resources.

Departing from the dynamic nature of culture, as defined by Marsella [31], for our results implies that in times of crises, people’s values, behaviors, and meanings of what are resourceful coping methods may change.

Our earlier study among academics in Iran showed that the two most frequent coping methods were life as part of a greater whole and praying to Allah/God [37]. These can be considered important meaning-making activities, and are not necessarily limited to either an individual or social level, but activities that have links to both, as they are shared with a larger community, context, or part of a system. What is more interesting is the result by Ahmadi et al. [37], showing that the individual dimension of meaning-making, being alone and contemplating to deal with crises, is highly ranked among middle-aged men. The two different aspects of meaning-making, individual and social, do not need to exclude each other. Rather, this reflects that the culture, which breeds the meaning-making process, includes both aspects, and they are both present and important in a society such as Iran. As Ungar pointed out, both processes of meaning-making, individual and macro system, are connected [22]. This is seen in our results where both individual- and community-related coping methods are ascribed importance, as well as structural factors, such as place of residence, distance-learning possibilities, and employment opportunities. With both obstacles and opportunities at hand, the Iranian population in our study shows an agency of balancing and making use of their social ecology based on their personal characteristics. An ability to balance inner and outer aspects of resources, and at times negotiate, is the strongest demonstration of resiliency and coping.

## 7. Conclusions

If we see resilience as a coping behavior reflecting positive outcomes in spite of a serious crisis, we may conclude that the Iranian population in our study shows a strong level of resilience, with both individual and community dimensions. In our study, we have avoided focusing only on the individual aspect of resilience, but instead highlighted the community level, as well as the influence of environment and structure, through different items. Importantly, being sensitive to the cultural and contextual dimensions of resilience, we have also avoided a normative approach to resilience, normalcy, and health. An interpretation of individual factors having dominance for resilience would have left us with an approach to resilience mainly dependent on the CD-RISC results. To balance this, we included items focused on social factors. These combined, the items better reflect the interdependency of cultural practices and give an important balance to the understanding of resilience. This study also shows clearly that a sharp distinction between individual and social dimensions of resilience and meaning-making is less useful in the Iranian context.

The strength of this study is the novelty of the early research on COVID-19, with a focus on resilience, coping, and health, especially so in a non-western context. The research in this area is still limited. Our study has contributed to the theoretical framework of resiliency by studying both individual factors of resilience and coping, as identified in the CD-RISC and coping instruments, as well as the social and cultural factors of meaning. By doing this, we avoid an oversimplification of resilience and pay more attention to real-world experiences and contextually specific factors for resiliency. We also, in line with Ni et al. [38], pay respect to the socio-cultural and socio-political factors present and relevant to resiliency.

As early explorative research, we may also suggest hypotheses for theory building:

**H1**  . *Among academic personnel in Iran and in times of crises, gender and age differences in resilience are limited.*

**H2.**  *Among academic personnel in Iran and in times of crises, men more than women express positive health.*

**H3.**  *Among academic personnel in Iran and in times of crises, family more than religion/spirituality gives life meaning.*

**H4.**  *Among academic personnel in Iran and in times of crises, there is a positive strong correlation between self-rated health and the use of different coping methods.*

**H5.**  *Among academic personnel in Iran and in times of crises, there is a significant correlation between resiliency and the use of different coping methods.*

### 7.1. Limitations

One limitation of the current study is the convenience sampling frame, with relatively small subgroups, making the level of representativeness and generalization limited. Additionally, some subgroups were underrepresented. Second, while the CD-RISC 2 is a good instrument for measuring the individual dimension of resilience, a validated instrument measuring the social and collective dimensions of resilience would be an important contribution to research. Third, we reached out specifically to academics, with the consequence that our results do not reflect other employed categories at universities. Statistical studies are useful for generalizing; however, to capture the experiences and processes of the meaning-making dimensions of resilience, in-depth interviews, and qualitative inquiries would be more appropriate and informative. Despite these limitations, our study is novel and may contribute to building our knowledge base on resilience in times of crisis.

### 7.2. Future Research and Policy Recommendations

Based on the results of this study, we suggest a few specific topics for future research:

- Future research should clearly focus on the ecological framework of resilience in the context of COVID-19, paying attention to individual, environmental, and meaning dimensions.
- This study conducted a simple analysis of univariate correlation (Pearson’s correlation). A multivariate analysis, such as regression analysis, adjusting for age, sex, and location, is required for future research to strengthen our findings.
- Qualitative and mixed-method studies provide better data for understanding the meaning-making processes in resilience. Such studies would add to the knowledge base on dealing with COVID-19 and its psychological consequences.

We would also like to suggest some recommendations that are relevant for policy or practice in times of crises:

- In clinical settings and during interventions for building and improving resilience in academic people after a pandemic, a holistic approach, including personal, micro, meso, and macro dimensions of resilience, may be more beneficial.
- Revise and adapt, in a contextual way, structural resources in society to better balance individual capacity resources for resilience.
- Academics can in times of crises benefit from adhering to individual and social dimensions of meaning-making, as well as searching for a balance between the obstacles and opportunities in their environment.

## Figures and Tables

**Table 1:** Respondents’ demographic characteristics (*n* = 196).

Variable		Frequency	Percentage
Gender	Male	49	25
	Female	147	75
Age	<25 years old	61	31
	25–35 years old	76	39
	>35 years old	59	30
Education	High school or similar	4	2
	University	192	98
Country of birth	Iran	194	99
	Afghanistan	2	1
Country of residence	Iran	194	99
	Switzerland	2	1
Work/student status	Full-time employment	49	25
	Part-time employment	27	14
	Campus student	43	22
	Distance-learning student	77	39
Civil status	Married	74	38
	Divorced	4	2
	Engaged	8	4
	Single	110	56
Children	Children	45	23
	No children	151	77
Place of residence	Capital	114	58
	Medium–large city	33	17
	Small town, close to a large city	37	19
	Small town, far from a large city	12	6

## Data Availability

The data presented in this study are available upon request from the corresponding author.
